# Outcomes and predictors of atrial fibrillation in cardiac amyloidosis

**DOI:** 10.1097/MD.0000000000046522

**Published:** 2026-04-03

**Authors:** Wajeeh ur Rehman, Imad Alam, Tariq Shah, Javaria Zahir, Muhammad Sulayman Khan, Hadiqa Khalil, Muhammad Osama, Waheed Akhtar, Jahanzeb Malik, Abida Parveen

**Affiliations:** a Department of Medicine, Ibn e Seena Hospital, Kabul, Afghanistan.

**Keywords:** AL-CA, anticoagulation, atrial fibrillation, cardiac amyloidosis, wild-type ATTR-CA

## Abstract

Cardiac amyloidosis (CA) frequently coexists with atrial fibrillation (AF), but the extent and impact of this relationship remain unclear. This study investigates the prevalence of AF in CA, compares clinical characteristics between amyloid light-chain (AL)-CA and amyloid transthyretin (ATTR)-CA, and assesses the implications for treatment strategies. We conducted a retrospective analysis of 108 patients with confirmed CA, categorizing them into 2 groups: 51 with AF and 57 with sinus rhythm (SR). We compared demographic, clinical, and echocardiographic data, as well as treatment modalities and outcomes between AL-CA and ATTR-CA subgroups. AF was present in 47% of the cohort, with a notably higher prevalence in wild-type ATTR-CA (80%) compared to AL-CA (33%). Patients with AF were older (73.8 ± 8.5 years) than those with SR (67.7 ± 10.6 years, *P* < .001) and had more severe heart failure symptoms, comorbidities, and echocardiographic abnormalities, including reduced left ventricular ejection fraction (LVEF; 47% vs 53%, *P* = .003) and higher pulmonary artery pressures (41 ± 11 mm Hg vs 35 ± 17 mm Hg, *P* = .02). Logistic regression identified BMI (OR 1.2, 95% CI: 1.05–1.57, *P* = .02) and eGFR (OR 0.91, 95% CI: 0.92–0.99, *P* = .04) as independent predictors of AF. CHA2DS2-VASc scores were higher in ATTR-CA patients (4.5 ± 1.2) compared to AL-CA (2.4 ± 1.1, *P* < .001). Despite a high rate of anticoagulation (78%), ischemic stroke or TIA incidence was low at 6% over 12 months. AF is highly prevalent in CA, particularly in ATTR-CA, and is associated with more severe clinical manifestations. The findings highlight the necessity for systematic AF screening and tailored management strategies in CA patients. Prospective, randomized studies are essential to establish optimal AF treatment protocols in this population.

## 1. Introduction

Cardiac amyloidosis (CA) has e merged as an important and often underdiagnosed cause of cardiomyopathy, characterized by the pathological deposition of amyloid proteins within the myocardium.^[1,2]^ The two major forms that commonly involve the heart are amyloid transthyretin (ATTR) and amyloid light-chain (AL) amyloidosis, each associated with distinct clinical courses and prognostic implications.^[3,4]^ While hereditary ATTR (hATTR) and AL amyloidosis are relatively rare, wild-type ATTR (wtATTR) is increasingly recognized as a more prevalent form, particularly among elderly patients and in individuals with heart failure or severe aortic stenosis.^[5-11]^

Infiltration of amyloid proteins affects not only the ventricles but also the atria, valves, and conduction system, leading to a wide spectrum of cardiac manifestations. These include atrial dysfunction, valvular abnormalities, conduction disturbances, and arrhythmias such as atrial fibrillation (AF).^[12-16]^ AF is particularly frequent in patients with CA and is associated with serious complications, including intracardiac thrombus formation and heightened thromboembolic risk, even in the absence of conventional predisposing factors.^[15,16]^ Consequently, timely identification of AF and consideration of anticoagulation therapy are critical aspects of disease management in amyloid cardiomyopathy.

In light of these considerations, the present study was undertaken to investigate the prevalence, clinical characteristics, and predictors of AF in newly diagnosed, treatment-naïve patients with AL or ATTR amyloidosis. By focusing on an untreated cohort, the study aims to provide insight into the natural presentation of AF in amyloid cardiomyopathy and to inform strategies for early risk assessment and management.

## 2. Materials and methods

### 2.1. Study design and population

This retrospective observational study included patients diagnosed with AL or ATTR cardiac amyloidosis (CA) at a tertiary care academic center between January 2015 and June 2024. Patients with confirmed CA and complete baseline clinical and echocardiographic data were eligible. Exclusion criteria were incomplete records, prior treatment for amyloidosis, or cardiomyopathy due to non-amyloid causes. Institutional review board approval was obtained (AIMS/24/70).

### 2.2. Diagnosis of cardiac amyloidosis

ATTR-CA was diagnosed by endomyocardial biopsy or noninvasive bone scintigraphy with technetium tracers (Perugini Grade 2–3 uptake) in the absence of plasma cell dyscrasia. Genetic sequencing of the transthyretin gene was performed for subtype classification. AL-CA was confirmed by histology from endomyocardial or surrogate tissue biopsy plus imaging findings consistent with amyloidosis.

### 2.3. Definition and classification of AF

Atrial fibrillation (AF) was defined per the 2020 ESC Guidelines as an irregular supraventricular tachyarrhythmia with uncoordinated atrial activity, documented by ECG or rhythm monitoring. Patients were classified as AF if arrhythmia was present at diagnosis or documented previously, regardless of subtype (paroxysmal, persistent, or permanent). ATTR staging followed the Gillmore system,^[17]^ while AL staging used the modified Mayo 2004 criteria.^[18]^

### 2.4. Echocardiography

Transthoracic echocardiography was performed at diagnosis using standard protocols. Chamber dimensions, systolic and diastolic function, and valvular lesions were assessed. Aortic stenosis was evaluated by continuity equation and mitral regurgitation by multiparametric methods. Global longitudinal strain (GLS) was calculated using speckle-tracking from apical views to assess myocardial deformation.

### 2.5. Statistical analysis

Continuous variables were reported as mean ± SD or median (IQR), and categorical variables as counts (percentages). Between-group comparisons used the *t*-test or Mann–Whitney *U* test for continuous variables and χ^2^ or Fisher’s exact test for categorical data. Trends between CA stage and AF prevalence were assessed with the Cochran–Armitage test. Multivariable logistic regression identified predictors of AF, with results presented as odds ratios (ORs) and 95% confidence intervals (CIs). A *P* < .05 was considered statistically significant. Analyses were performed using SPSS version 23.0 (IBM Corp., Armonk).

## 3. Results

The study included 108 patients diagnosed with CA, of which 51 had AF and 57 were in sinus rhythm (SR). Patients with AF were significantly older on average compared to those in SR (73.8 years vs 67.7 years, *P* < .001) and had a higher proportion of males (90% vs 68%, *P* = .01; Table 1). The distribution of CA types differed significantly between AF and SR groups, with a higher prevalence of AL amyloidosis among those in SR (72% vs 33%, *P* < .001).

**Table 1:** Patient characteristics.

Variable	Overall (n = 108)	AF (n = 51)	SR (n = 57)	*P* value
Age	69.2 ± 11.8	73.8 ± 8.5	67.7 ± 10.6	<.001
Male	85 (79)	46 (90)	39 (68)	0.01
CA type				
AL	58 (54)	17 (33)	41 (72)	<.001
Hereditary ATTR	6 (6)	1 (2)	5 (9)	
Wild-type ATTR	44 (41)	33 (65)	11 (19)	
Body mass index (kg/m^2^)	25.4 ± 5.8	24.9 ± 3.5	25.8 ± 7.2	.47
NYHA class				
I/II	49 (45)	19 (37)	30 (53)	.07
III/IV	59 (55)	32 (63)	27 (47)	
Comorbidities				
Arterial hypertension	50 (46)	28 (55)	22 (39)	.12
Chronic kidney disease	47 (44)	29 (57)	18 (32)	.03
Diabetes	20 (19)	14 (27)	6 (11)	.06
COPD	4 (4)	1 (2)	3 (5)	.61
Coronary artery disease	35 (32)	25 (49)	10 (18)	<.001
Stroke	10 (9)	3 (6)	7 (12)	.22
Dyslipidaemia	27 (25)	18 (35)	9 (16)	.03
Anaemia	52 (48)	26 (51)	26 (46)	.63
Pacemaker	11 (10)	8 (16)	3 (5)	.13
Laboratory analysis				
NT-proBNP (pg/mL)	4214 (8110)	5883 (9234)	2587 (9685)	.07
Haemoglobin (g/dL)	12.3 ± 2.0	11.8 ± 2.2	12.0 ± 1.8	.43
eGFR (mL/min/1.73 m^2^)	56 ± 23.1	48.1 ± 21.8	59.2 ± 27.8	.37
Creatinine (mg/dL)	1.5 (1.3)	1.6 (0.8)	1.5 (0.8)	.36
Leucocyte count	7.9 ± 3.3	7.6 ± 2.1	8.4 ± 4.8	.83
C-reactive protein (mg/dL)	0.2 (1.2)	0.1 (1.4)	0.3 (1.5)	.18

AF = atrial fibrillation, AL = amyloid light-chain, ATTR = amyloid transthyretin, CA = cardiac amyloidosis, COPD = chronic obstructive pulmonary disease, eGFR = estimated glomerular filtration rate, NT-proBNP = N-terminal pro–B-type natriuretic peptide, NYHA = New York Heart Association, SR = sinus rhythm.

In terms of comorbidities, patients with AF had higher rates of chronic kidney disease (57% vs 32%, *P* = .03), diabetes (27% vs 11%, *P* = .06), coronary artery disease (49% vs 18%, *P* < .001), and dyslipidemia (35% vs 16%, *P* = .03) compared to those in SR. There were no significant differences in body mass index, N-terminal pro–B-type natriuretic peptide levels, or renal function (eGFR) between the two groups.

Echocardiographic findings revealed that patients with AF had significantly lower left ventricular ejection fraction (LVEF) compared to those in SR (47% vs 53%, *P* = .003) and tended to have higher tricuspid regurgitation velocity (TRV; 2.8 m/s vs 2.5 m/s, *P* = .04) and systolic pulmonary artery pressure (systolic PAP; 41 mm Hg vs 35 mm Hg, *P* = .02; Table 2). There were no significant differences in other echocardiographic parameters including global longitudinal strain (GLS), left ventricular mass index (LVMMI), or left atrial volume index (LAVI).

**Table 2:** Echocardiographic findings.

Variable	Overall (n = 108)	AF (n = 51)	SR (n = 57)	*P* value
LVEF (%)	50 ± 11	47 ± 12	53 ± 9	.003
GLS (%)	10.5 ± 4.3	10.1 ± 3.7	10.8 ± 4.7	.37
IVSd (mm)	15 ± 5	16 ± 5	15 ± 5	.43
PWd (mm)	15 ± 6	14 ± 4	15 ± 5	.19
LVEDD (mm)	48 ± 8	47 ± 9	49 ± 7	.27
LVMMI (g/m^2^)	170 ± 47	167 ± 47	173 ± 46	.12
LAVI (mL/m^2^)	52 ± 18	54 ± 18	50 ± 19	.09
e′ (cm/s)	6.7 ± 2.3	6.3 ± 1.9	7.1 ± 2.6	.06
E/e′	16 ± 7	17 ± 8	15 ± 6	.11
TRV (m/s)	2.6 ± 0.5	2.8 ± 0.5	2.5 ± 0.6	.04
Systolic PAP (mm Hg)	38 ± 14	41 ± 11	35 ± 17	.02
TAPSE (mm)	19 ± 6	18 ± 6	20 ± 6	.08
RV free wall thickness (mm)	7.1 ± 2.6	6.8 ± 2.2	7.4 ± 3.0	.19
Pericardial effusion	24 (18)	13 (25)	11 (19)	.41
Mitral regurgitation ≥ Grade 2	49 (45)	32 (63)	17 (30)	<.001
Aortic stenosis ≥ Grade 2	3 (3)	1 (2)	2 (4)	.67

AF = atrial fibrillation, GLS = global longitudinal strain, IVSd = interventricular septal thickness in diastole, LAVI = left atrial volume index, LVEDD = left ventricular end-diastolic diameter, LVEF = left ventricular ejection fraction, LVMMI = left ventricular mass index, PAP = pulmonary artery pressure, PWd = posterior wall thickness in diastole, RV = right ventricle, SR = sinus rhythm, TAPSE = tricuspid annular plane systolic excursion, TRV = tricuspid regurgitation velocity.

Regarding the association between AF and the stage of CA, patients with ATTR-CA in stage III had the highest prevalence of AF compared to those in stage I and stage II (*P* < .001). In contrast, there was no significant linear trend observed between AF and stages of AL-CA (*P* < .001; Table 3).

**Table 3:** Linear trend between AF and stage of cardiac amyloidosis.

ATTR-CA stage	(n = 19)	*P* value
Stage I	13 (68)	<.001
Stage II	4 (21)	
Stage III	2 (11)	
AL-CA stage	AF (n = 3)	
Stage I	0 (0)	<.001
Stage II	1 (33)	
Stage IIIa	2 (67)	
Stage IIIb	0 (0)	

AF = atrial fibrillation, AL = amyloid light-chain, ATTR = amyloid transthyretin, CA = cardiac amyloidosis.

Logistic regression analysis showed that higher body mass index (OR 1.2, 95% CI: 1.05–1.57, *P* = .02) and lower eGFR (OR 0.91, 95% CI: 0.92–0.99, *P* = .04) were associated with increased odds of AF, while AL type CA was associated with lower odds of AF (OR 0.1, 95% CI: 0.01–0.3, *P* = .001; Table 4).

**Table 4:** Logistic regression for the likelihood of AF.

Variable	Odds ratio	95% CI	*P* value
Body mass index	1.2	1.05, 1.57	.02
eGFR	0.91	0.92, 0.99	.04
AL type	0.1	0.01, 0.3	.001

AF = atrial fibrillation, AL = amyloid light-chain, eGFR = estimated glomerular filtration rate.

In terms of treatment modalities for AF in CA patients, those with AL-CA and ATTR-CA received similar proportions of pharmacologic treatments such as anticoagulation (71% vs 85%) and beta-blockers/calcium channel blockers (63% vs 63%; Table 5). There were no significant differences in non-pharmacologic treatments or adverse clinical events between AL-CA and ATTR-CA groups.

**Table 5:** Characteristics and treatment modalities of atrial fibrillation in patients with cardiac amyloidosis.

Variable	Overall (n = 51)	AL-CA (n = 24)	ATTR-CA (n = 27)	*P* value
Age	74 ± 8	66 ± 9	79 ± 6	<.001
Male	41 (80)	12 (50)	29 (74)	.02
CHA2DS2-VASc score	3.8 (1.4)	2.4 (1.1)	4.5 (1.2)	<.001
EHRA symptom severity scale				
I	17 (33)	6 (25)	11 (41)	.12
IIa/b	22 (43)	11 (46)	11 (41)	
III	12 (24)	7 (29)	5 (18)	
IV	0 (0)	0 (0)	0 (0)	
AF burden				
Permanent	20 (39)	7 (29)	13 (48)	.09
Persistent	16 (31)	8 (33)	8 (30)	
Paroxysmal	15 (30)	9 (38)	6 (22)	
Pharmacologic treatments				
Anticoagulation	40 (78)	17 (71)	23 (85)	.17
Vitamin K antagonist	10 (20)	5 (21)	5 (19)	
Direct oral anticoagulant	30 (59)	12 (50)	18 (67)	
Digitalis glycosides	6 (12)	3 (13)	3 (11)	.72
Beta-blocker/calcium channel blocker	32 (63)	15 (63)	17 (63)	1
Amiodarone	4 (8)	2 (8)	2 (7)	1
Non-pharmacologic treatments				
Pacemaker	9 (18)	4 (17)	5 (19)	1
Current cardioversion	13 (25)	6 (25)	7 (26)	1
Catheter ablation	7 (14)	3 (13)	4 (15)	1
Adverse clinical events				
Bleeding	8 (16)	3 (13)	5 (19)	.61
Stroke/TIA	3 (6)	1 (4)	2 (7)	1

AF = atrial fibrillation, AL = amyloid light-chain, ATTR = amyloid transthyretin, CA = cardiac amyloidosis, TIA = transient ischemic attack.

## 4. Discussion

Our study demonstrates that nearly half of patients with AL or ATTR CA present with AF, a prevalence far exceeding that reported in the general population or in large heart failure trials.^[8-10,15,19,20]^ In particular, the prevalence of AF among wild-type ATTR-CA (wtATTR-CA) patients was remarkably high in our cohort, aligning with but also reaching the upper range of previously reported rates (40–88%).^[21,22]^ This observation strengthens the notion that AF is intrinsically linked to the pathophysiology of CA rather than merely a comorbid condition.

Compared with prior reports, our findings provide additional insights into the structural and functional basis of AF in CA. Histopathological and imaging studies have described diffuse amyloid infiltration of both atria and ventricles, altering conduction properties and atrial compliance.^[23,24]^ Consistent with this, patients with AF in our cohort had more severe clinical and echocardiographic abnormalities, including impaired biventricular function, elevated pulmonary pressures, and higher rates of mitral regurgitation.^[25]^ These results extend earlier work by confirming that AF in CA is not only common but also associated with a more advanced hemodynamic profile and greater comorbidity burden, including chronic kidney disease and coronary artery disease.

A novel contribution of this study is the differential association between AF prevalence and disease stage across amyloidosis subtypes. We observed a nearly universal prevalence of AF (96%) in stage III ATTR-CA, supporting a progressive relationship between disease severity and arrhythmia development. In contrast, AF prevalence declined with advancing stage in AL-CA, a finding that diverges from prior assumptions and highlights possible differences in disease mechanisms. While ATTR staging incorporates both biomarkers and renal function, AL staging relies exclusively on cardiac biomarkers, which may not fully reflect atrial remodeling and arrhythmic risk.^[25-27]^ This underscores the need for earlier AF surveillance in AL-CA, even at lower stages.

Our regression analysis further identified body mass index (BMI), estimated glomerular filtration rate (eGFR), and AL amyloidosis subtype as independent predictors of AF. This builds on earlier studies that have emphasized left atrial size or wall stress as contributors to AF,^[15]^ by introducing metabolic and renal function parameters as additional determinants. These findings suggest that AF in CA is multifactorial, shaped by both amyloid infiltration and systemic comorbidities.

With regard to management, most patients in our cohort were treated with anticoagulation and rate-controlling agents, reflecting guideline-based care. However, the effectiveness of rhythm control strategies, including cardioversion and catheter ablation, remains questionable in this population given the high recurrence rates reported in previous studies.^[25,28]^ Our findings reinforce this uncertainty and suggest that therapeutic decisions should be individualized, balancing thromboembolic prevention against the limited success of rhythm interventions.

Overall, this study contributes to the literature by: confirming the high prevalence of AF in CA, especially wtATTR-CA, highlighting stage-dependent differences between ATTR and AL subtypes, identifying novel predictors of AF such as BMI and eGFR, and raising important considerations regarding AF management in amyloidosis. These insights expand upon prior work and suggest that AF may not only be a marker of advanced disease but also a potential driver of progression. Future prospective studies are needed to clarify its prognostic impact and to optimize management strategies tailored to amyloidosis patients.

## 5. Limitations

The primary limitation of our study is its retrospective nature, which restricts the ability to conduct unbiased analyses of critical clinical questions such as the burden of arrhythmias, the effectiveness of different treatments for AF, and the precise temporal relationship between AF and CA. Retrieving data from digital medical records may introduce inherent biases and limitations in data completeness and accuracy. Additionally, our study was constrained by the fact that standardized evaluation and systematic follow-up protocols for CA patients were only established at our institution in 2018. As a result, not all patients underwent routine Holter monitoring or continuous rhythm assessment before this period, potentially leading to an underestimation of occult AF cases. Despite these limitations, the study’s strength lies in its extensive and detailed characterization of a large contemporary cohort of patients. This comprehensive phenotypic analysis provides valuable insights into the clinical presentation, comorbidities, and echocardiographic findings associated with AF in patients with CA. Future prospective studies with robust follow-up protocols and broader use of continuous rhythm monitoring are needed to further elucidate the impact of AF on CA outcomes and refine management strategies accordingly.

## 6. Conclusion

Our study underscores the significant prevalence of AF in patients with CA, particularly emphasizing the need for systematic AF screening, especially in those with advanced stages of ATTR-CA. Patients diagnosed with AF in our cohort exhibited more severe symptoms, poorer cardiac function, and a higher incidence of concurrent health conditions. Importantly, the occurrence of ischemic stroke or transient ischemic attack (TIA) within 12 months after diagnosis was relatively low, despite a high rate of anticoagulation therapy. The optimal management approach for AF in the context of CA remains a critical and unresolved clinical challenge. Prospective studies employing randomized designs are necessary to determine the most effective treatment strategies for AF in CA patients. These studies will provide clearer insights into how best to manage AF in this complex patient population, aiming to improve outcomes and quality of life through evidence-based interventions.

## Author contributions

**Conceptualization:** Wajeeh ur Rehman, Imad Alam, Tariq Shah, Javaria Zahir, Muhammad Sulayman Khan, Hadiqa Khalil, Muhammad Osama, Waheed Akhtar, Jahanzeb Malik, Abida Parveen.

**Data curation:** Javaria Zahir.

**Supervision:** Imad Alam.

**Writing – original draft:** Tariq Shah.

**Writing – review & editing:** Wajeeh ur Rehman, Imad Alam, Javaria Zahir, Muhammad Sulayman Khan, Hadiqa Khalil, Muhammad Osama, Waheed Akhtar, Jahanzeb Malik, Abida Parveen.
